# Effect of Citrullus colocynth fruit extract on pro and anti apoptotic molecules in human melanoma cell line (A375)

**DOI:** 10.6026/97320630018299

**Published:** 2022-03-31

**Authors:** Sariga Jayachandran, Preetha Santhakumar, Selvaraj Jayaraman, G Sridevi

**Affiliations:** 1 Department of physiology, Saveetha Dental College and Hospitals, Saveetha Institute of Medical and Technical Sciences, Saveetha University, Chennai-600077, Tamil Nadu, India

**Keywords:** Citrullus colocynthis, anticancer therapy, anti apoptosis, melanoma, innovative technique

## Abstract

Melanoma remains a major cause of death worldwide. Chemotherapy might be used to treat advanced Melanoma which is associated with some side effects. Hence, providing proper treatment or proper employment of lower toxic substances is essential. Citrullus colocynthis, a medicinal plant, seems a potential anticancer herbal medicine against Melanoma via various efficient compounds. Therefore, it is of interest to analyze the effect of Citrullus colocynthis fruit extract on pro and anti apoptotic molecules in the human melanoma cell line (A375). Cell viability test was done using MTT assay. mRNA expression of Bax and Bcl2 was done by real-time PCR. The data was analyzed statistically by one way analysis of variance and Duncan multiple range test with graph prism version 5. p<0.05 was considered significant. Citrullus colocynthis extract caused a marked increase in cell death in a dose-dependent manner. At the end of 48 hours, maximum inhibition (50%) was at 400 and 500µg/ml. The study demonstrated that the effect of Citrullus colocynthis extract has elevated the Bax mRNA expression on human melanoma cell lines. Data shows that Citrullus colocynthis fruit extract has an anticancer effect on the human melanoma cell line (A375) by increasing the expression of Bax and Bcl2 and this could be because of the phytochemicals present in it.

## Background:

Citrullus colocynthis has been reported to possess a wide range of traditional medicinal uses in treatment of diseases like diabetes, leprosy, cold, cough, jaundice, joint pain, cancer, toothache, wound, and in gastrointestinal disorders like indigestion, constipation, dysentery, gastroenteritis, colic pain and different microbial infections [1]. Several bioactive chemical constituents from fruits were recorded, such as, glycosides, flavonoids, alkaloids, fatty acids and essential oils. The isolation and identification of cucurbitacins were also reported in Citrullus colocynthis [2]. The plant was also shown to be rich in nutritional value with high protein contents and important minerals also as an edible quality of seed oil [3]. Cancer constitutes a group of deadly diseases that's not only the second leading explanation for death worldwide, but also largely contributes to the worldwide health economic burden [4]. There is a need to design novel drugs with high efficacy specific for cancer cells and less toxicity [5]. New targeted therapies, like BRAF and immune checkpoint inhibitors, have achieved success in extending patient survival, however, innate or acquired therapy resistance and tumour recurrence is nearly unavoidable [6]. Although most melanomas develop on the skin, they will originate in almost any organ including brain and lymph nodes. Melanomas are often cured with relative operation. Melanomas are often more serious than the opposite sorts of carcinoma because it tends to spread (metastasis) to other parts of the body, causing serious illness and death [7]. Medicinal plants are regarded as a treasure house of drugs [8-9 - check with author]. Due to the strong therapeutic effects, the medicinal plants have been traditionally used to treat diseases [10-12]. Different parts of medicinal plants have numerous nutraceutical values and are enriched with proteins, carbohydrates, vitamins, fibre, potassium, calcium and also the presence of phytoconstituents contributes to its significant medicinal property [13 - check with author, 15]. Our team has extensive knowledge and research experience that has translated into high quality publications [16-36]. Therefore it is of interest to analyze the effect of Citrullus colocynthis fruit extract on pro and anti apoptotic molecules in human melanoma cell lines (A375).

## Materials and methods:

Dimethyl sulfoxide (DMSO), 3-(4,5-dimethylthiazol-2-yl)-2,5-diphenyltetrazolium bromide (MTT) were purchased from Sigma Chemical Pvt Ltd, USA. Trypsin-EDTA, fetal bovine serum (FBS), antibiotics-antimycotics, RPMI 1640 medium and phosphate buffered saline (PBS) were purchased from Gibco, Canada. (5,5,6,6-tetrachloro-1,1,3,3 -tetraethyl benzimidazolo carbocyanine iodide) and Real Time PCR kit was purchased TAKARA (Meadowvale Blvd, Mississauga, ON L5N 5S2, Canada).

## Cell lines and cell culture:

Human melanoma cancer cell line (A375) was purchased from National Centre for Cell Sciences (NCCS), Pune, India. Cells were cultured in DMEM medium (Thermo Fisher Scientific, CA, USA) containing 10% fetal bovine serum (Thermo Fisher Scientific, CA, USA), 100 U/ml penicillin and 100 µg/ml streptomycin (Thermo Fisher Scientific, CA, USA) at 37°C with 5% CO_2_.

## Cell viability by MTT assay:

Cell viability was assayed using a modified colorimetric technique that is based on the ability of live cells to convert MTT, a tetrazolium compound into purple formazan crystals by mitochondrial reductases (Mosmann, 1983). Briefly, the cells (1 x 104/well) were exposed to different concentrations of Citrullus colocynthis fruit extract (100-500µg/ml) with A375 cells for 48 h. At the end of the treatment, 100 µl of 0.5 mg/ml MTT solution was added to each well and incubated at 37°C. Then the formazan crystals formed were dissolved in dimethyl sulfoxide (100 µl) and incubated in dark. Then the intensity of the color developed was assayed using a Micro ELISA plate reader at 570 nm. The number of viable cells was expressed as the percentage of control cells cultured in the extract free medium. Cell viability in the control medium without any treatment was represented as 100%. The cell viability is calculated using the formula: % cell viability = [A570 nm of treated cells/A570 nm of control cells] x 100.

## Gene expression analysis by Real Time-PCR:

Samples from each group were submerged in 2 ml Trizol (Invitrogen, Carlsbad, CA, USA) for RNA extraction and stored at -80°C until further processed. cDNA synthesis was performed on 2 µg RNA in a 10 µl sample volume using Superscript II reverse transcriptase (Invitrogen) as recommended by the manufacturer. Real-time PCR array analysis was performed in a total volume of 20 µl including 1 µlcDNA, 10 µlqPCR Master Mix 2x (Takara, USA) and 9 µl ddH2O. Reactions were run on an CFX96 Touch Real-Time PCR Detection System (Bio-Rad, USA) using universal thermal cycling parameters (95°C for 5 min, 40 cycles of 15 sec at 95°C, 15 sec at 60°C and 20 sec at 72°C; followed by a melting curve: 5 sec at 95°C, 60 sec at 60°C and continued melting). For quality control purposes, melting curves were acquired for all samples. The specificity of the amplification product was determined by melting curve analysis for each primer pair. The data were analyzed by comparative CT method and the fold change is calculated by 2−ΔΔCT method described by Schmittgen and Livak (2008) using CFX Manager Version 2.1 (Bio Rad, USA).

## Statistical analysis:

The obtained data were analyzed statistically by one-way analysis of variance (ANOVA) and Duncan's multiple range tests with computer-based software (Graph Pad Prism version 5) to analyze the significance of individual variations among the control and experimental groups. The significance was considered at p<0.05 level in Duncan's test.

## Results:

### Effect of Citrullus colocynthis on cell viability in A375 cells:

Citrullus colocynthis was evaluated against human melanoma cancer line (A375) and cell viability was determined after administering the different doses of Citrullus colocynthis fruit extract. It was found to exhibit inhibition of cancer cells by decreasing the percent viability of cancer cells in a dose-dependent manner. However, 400-500 µg/ml concentration of the extract showed maximum inhibition of the viability of the human melanoma cells suggesting that Citrullus colocynthis induces apoptosis in A375 Cells (Figure 1 - see PDF).

### Effect of Citrullus colocynthis on Bcl2 mRNA expression in A375 cells:

In comparison with untreated control cells, Bcl2 mRNA expression was found to be significantly decreased (p<0.05). In groups treated with 300 and 400 µg/ml concentration of Citrullus colocynthis extract, expression of Bcl2 mRNA is reduced when compared to the untreated control group (Figure 2 - see PDF).

### Effect of Citrullus colocynthis on Bax mRNA expression in A375 cells:

In comparison with untreated control cells, Bax mRNA expression was found to be significantly increased (p<0.05). In groups treated with 300 and 400 µg/ml concentration of Citrullus colocynthis extract, expression of Bax mRNA is increased when compared to untreated control group (Figure 3 - see PDF).

## Discussion:

The present study was carried out to analyze the effect of Citrullus colocynthis fruit extract on human melanoma cell line (A375). Citrullus colocynthis is an annual or a perennial plant which grows in sandy, arid soils. Various studies were done on this plant to predict the restorative compatibility of the plant. Citrullus colocynthis, an important plant, is well known for its medicinal properties. Different parts of this plant are used traditionally in the treatment of disease conditions such as diabetes, high cholesterol and blood fats called triglycerides, constipation and tuberculosis [1]. Apoptosis is the mechanism for the anti-proliferative effect of neoplastic cells. Mitochondria- initiated responses are involved in regulation of the intrinsic apoptotic pathway. The metabolic activities of mitochondria in cancer cells are distinct from the normal cells. The metabolic activities of mitochondria in cancer cells are considered to be a biologically significant source of apoptotic failure that is closely in association with chemotherapy [4]. It has been hypothesized that Bax induces the discharge of cytochrome by inhibiting Bcl-2 function through binding of the BH1, BH2, and BH3 domains. There is, however, considerable evidence to support the hypothesis that Bax and Bcl-2 function independently in regulating apoptosis [36]. Both Bcl-2 and Bax are capable of forming ion channels in artificial membranes, although Bcl-2 inhibits the activity of Bax at neutral pH [36]. These data would suggest that although the functions of Bax are often inhibited by Bcl-2 the removal of some of the BH1, BH2, and BH3 domains don't prevent Bax from enhancing chemotherapy-induced apoptosis. Likewise, Bax mutant proteins that fail to bind to Bcl-2 are capable of inducing apoptosis [37]. Previous studies on Bcl2 and Bax have shown that Bcl2 and Bax are involved in the control of apoptotic pathways. The ratio between Bcl2 and Bax represents a cell rheostat that is able to predict a cell's response to an apoptotic stimulus. The methanolic extract of Citrullus colocynthis fruit extract has shown significant anticancer potential against breast cancer cell line MCF-7 in a dose dependent manner. Previous studies done on this plant have reported that it has a cytotoxic effect on breast cancer cell lines which are similar to our study [37]. Further studies can be carried out in the future in Citrullus colocynthis to exactly find out about the underlying mechanisms.

## Conclusion:

Data showed that the fruit extract of Citrullus colocynthis has cytotoxic effect against human melanoma cell lines through inhibition of Bcl2 mRNA expression or by elevating the Bax mRNA expression on human melanoma cell lines (A375) and it also plays a role in prevention of cell proliferation, induction of cell apoptosis and inhibition of cancer stemness property on human melanoma cells.

## Source of funding:

This study is funded by Saveetha institute of medical and technical sciences, Saveetha dental college, Saveetha University, Balakrishna nursery and primary school.
